# A Genome-Wide Association Study of the Metabolic Syndrome in Indian Asian Men

**DOI:** 10.1371/journal.pone.0011961

**Published:** 2010-08-04

**Authors:** Delilah Zabaneh, David J. Balding

**Affiliations:** Department of Epidemiology and Public Health, Imperial College London, London, United Kingdom; Karolinska Institutet, Sweden

## Abstract

We conducted a two-stage genome-wide association study to identify common genetic variation altering risk of the metabolic syndrome and related phenotypes in Indian Asian men, who have a high prevalence of these conditions. In Stage 1, approximately 317,000 single nucleotide polymorphisms were genotyped in 2700 individuals, from which 1500 SNPs were selected to be genotyped in a further 2300 individuals. Selection for inclusion in Stage 1 was based on four metabolic syndrome component traits: HDL-cholesterol, plasma glucose and Type 2 diabetes, abdominal obesity measured by waist to hip ratio, and diastolic blood pressure. Association was tested with these four traits and a composite metabolic syndrome phenotype. Four SNPs reaching significance level p<5×10^−7^ and with posterior probability of association >0.8 were found in genes CETP and LPL, associated with HDL-cholesterol. These associations have already been reported in Indian Asians and in Europeans. Five additional loci harboured SNPs significant at p<10^−6^ and posterior probability >0.5 for HDL-cholesterol, type 2 diabetes or diastolic blood pressure. Our results suggest that the primary genetic determinants of metabolic syndrome are the same in Indian Asians as in other populations, despite the higher prevalence. Further, we found little evidence of a common genetic basis for metabolic syndrome traits in our sample of Indian Asian men.

## Introduction

The metabolic syndrome is the combination of most or all of: raised plasma glucose, abdominal obesity, dyslipidemia, and high blood pressure [1]. People affected by the metabolic syndrome are at an increased risk of coronary heart disease and type 2 diabetes (T2D), which are large and rapidly-increasing causes of illness and death globally. Indian Asians have a high prevalence of the metabolic syndrome compared with Europeans, and metabolic syndrome traits are highly heritable in Indian Asians (h^2^ between 0.27and 0.53 [2]). It is thought that the syndrome results from a complex interplay of genetic and environmental factors, and genetic variants underlying metabolic traits have been identified at several loci in European-origin populations. Little is known about whether the same genetic mechanisms trigger metabolic disturbances in Indian Asians as in Europeans, nor whether there are genetic mechanisms that are common across metabolic syndrome traits in Indian Asians. Our study was intended to investigate these two questions.

We screened common SNPs for association with metabolic syndrome and four of its component traits in a sample of Indian Asian men. Metabolic syndrome traits vary substantially between men and women; to reduce heterogeneity we included only men in this study. The four traits included three quantitative traits (diastolic blood pressure DBP, waist-hip ratio WHR, and HDL cholesterol), and one binary (presence or absence of T2D). We also tested a metabolic syndrome phenotype that was created from individual metabolic traits. We used a two-stage design, with independent “top and tail” sample selection and different genotyping platforms in each stage. Overall, about 1500 SNPs, primarily selected from the Stage 1 results, were genotyped in approximately 5000 individuals in the two stages combined. We report p-values of association under an additive model, after adjustment for covariates (see Materials and Methods). Although familiar, p-values suffer from problems of interpretation and the difficulty of combining signals under different genetic models [3]. We therefore also report the posterior probability of association (PPA) for a 4∶1 weighting of additive and general genetic models (see Materials and Methods). We assumed a prior probability of association of 10^−4^ for each trait, which corresponds to a cautious assumption that only around 300 kb of the genome is in high linkage disequilibrium (LD) with a causal variant. The PPA is a directly interpretable measure of weight of evidence for association, and gives due emphasis to additive genetic models while also allowing strong, non-additive signals of association to be taken into account.

## Results

**Figure 1:**
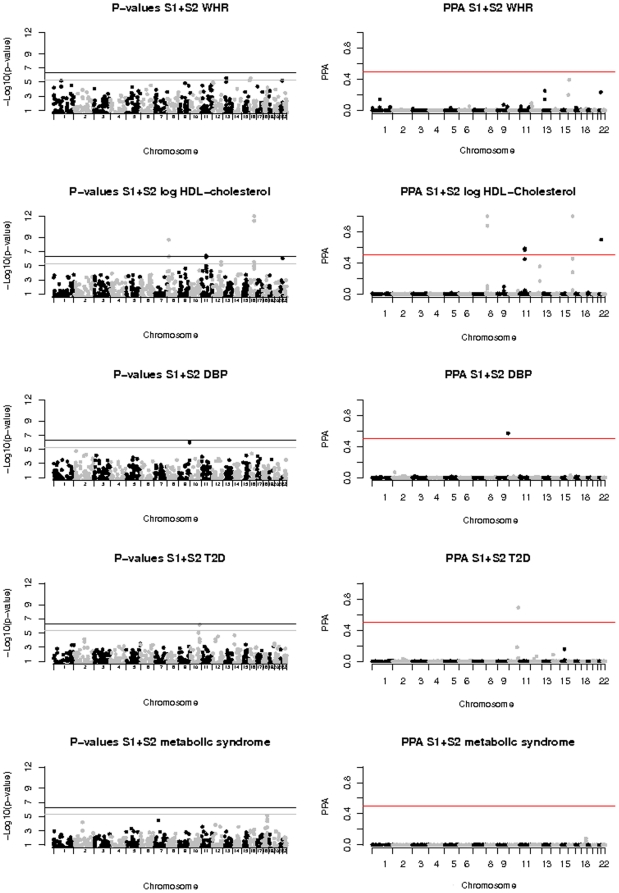
Genome-wide association results of the combined Stage 1 and Stage 2 analysis.

Characteristics of individuals selected for genotyping in each stage are shown in Table 1. Quantile-quantile (Q-Q) and signal intensity plots for Stage 1 results are in Figure S1. In summary, after combining data from stages 1 and 2 (Figure 1), four SNPs at two loci were strongly associated with HDL-cholesterol (p<5×10^−7^, PPA>0.8). In addition, a TCF7L2 SNP had a PPA of almost 0.7 for T2D (p = 7×10^−7^). A further four SNPs were associated with HDL or DBP at p<10^−6^ and PPA>0.5. These results are further described below and in Table 2, and a list of all SNPs significant at p<10^−5^ from the combined analysis is in Table S1.

**Table 1:** Characteristics of genotyped Indian Asian men.

	Stage 1 (N = 2684)		Stage 2 (N = 2020)		Combined (N = 4560)	
Description	Mean (SD) or prevalence	Range	Mean (SD) or prevalence	Range	Mean (SD) or prevalence	Range
**Age** (yrs)	50.0 (11.0)	35.0–74.8	47.5 (10.7)	35.0–74.8	49.9 (10.9)	35.0–74.8
**CHD** (%)	8%	-	8%	-	8%	-
**Hypertension** (%)	33%	-	33%	-	33%	-
**T2D** (%)	25%	-	25%	-	25%	-
**Cholesterol med** (%)	18%	-	17%	-	18%	-
**SBP** (mmHg)	134.2(20.6)	87–231	133.8 (19.9)	89–244	134.1 (20.4)	87–244
**DBP** (mmHg)	82.6 (12.1)	53–132	83.0 (11.9)	55–149	82.8 (12.0)	53–149
**WHR**	0.97 (0.07)	0.55–1.29	0.97 (0.07)	0.68–1.41	0.97(0.07)	0.55–1.41
**HDL-cholesterol** (mmol/l)	1.22 (0.31)	0.57–3.28	1.22 (0.33)	0.25–4.85	1.22(0.32)	0.25–4.85
**Glucose** (mmol/l)	6.03 (2.18)	2.60–21.90	6.04(2.20)	2.00–21.40	6.04(2.19)	2.00–21.9
**Metabolic syndrome** (IDF)	47.0%	-	49.0%	-	48.0%	-

Prevalence of the metabolic syndrome is for the selected sample and is not representative of the general population.

**Table 2:** SNPs with p-value <10^−6^ and PPA >0.5.

Combined data (N = 4794)												Reported associations		
Phenotype	SNP	Locus	Position	Minor allele	MAF	Effect/OR (95% CI)	Log10 BF‡PPA G/A	P	% variance explained	Nearest Gene	Previous reports*	Reported MAF	Reported p-value	Ref
**Log HDL-C (mmol/l)**	rs3764261	16q13	55,550,824	A	0.36	0.07 (0.06 to 0.08)	40/43 1.00	1.3×10^−48^	4.60	CETP	EU, IA	0.31	2×10^−18^	[6], [8], [24], [25]
**“**	rs9989419	16q13	55,542,639	A	0.39	−0.05 (−0.05 to −0.04)	15/17 1.00	1.4×10^−20^	1.90	CETP	EU, IA	0.35	3×10^−31^	[24]
**“**	rs2083637	8p21.3	19,909,454	G	0.24	0.04 (0.02 to 0.05)	5.9/7.4 1.00	1.9×10^−10^	0.89	LPL	EU, IA	0.26	6×10^−18^	[26]
**“**	rs4523270	8p21.3	19,900,818	G	0.27	0.03 (0.02 to 0.04)	3.5/4.9 0.88	1.0×10^−07^	0.56	LPL	EU, IA	a	a	a
**“**	rs496300	21q22.3	43,604,107	G	0.16	0.03 (0.02 to 0.05)	3.0/4.5 0.70	3.9×10^−07^	0.56	FLJ41733	None	-	-	-
**“**	rs174546	11q12.2	61,326,405	A	0.18	−0.03 (−0.04 to −0.02)	3.1/4.2 0.58	6.0×10^−07^	0.55	FADS1	EU	0.44	1×10^−7^	[8]
**“**	rs1535	11q12.2	61,354,547	G	0.18	−0.03 (−0.04 to −0.02)	3.1/4.2 0.57	6.5×10^−07^	0.55	FADS2	EU	a	a	a
**T2D**	rs7903146	10q25.2	114,748,338	A	0.30	1.33 (1.19 to 1.49)	4.1/4.4 0.69	6.6×10^−07^	-	TCF7L2	EU, IA	0.44	2×10^−34^	[9]–[15] **
**DBP** (mmHg)	rs7865146	9q34.11	129,659,457	A	0.37	−1.19 (−1.67 to −0.71)	3.4/4.2 0.57	1.0×10^−06^	0.51	ENG	None	-	-	-

Alleles are from the “Top-Bottom” strand as provided by Illumina. Effect size shown is regression coefficient, which represents per-allele change in phenotype mean (after adjustment).

‡ Log10 Bayes Factor; G = General model, A =  additive model. PPA =  posterior probability of association, assuming prior 10^−4^. PPA was calculated from additive and general model BFs with 4∶1 weighting respectively.

*EU =  reported in Europeans, IA = reported in Indian Asians, None =  not reported.

a =  in high LD with rs174546, b =  no information available.

**Values given here are from [12].

Two SNPs in the FTO gene, which has a well-established association with obesity (e.g. [4]), were genotyped in our study (rs8050136 and rs3751812), and showed only weak association with WHR (p = 4.6×10^−3^ and 4.4×10^−3^). No SNP showed strong association with the compound metabolic syndrome phenotype. The largest PPA for a SNP associated with the metabolic syndrome as defined by the IDF [1] was 0.08 (p = 6.8×10^−6^) at rs12957347, about 180 kb upstream from gene PMAIP1 (phorbol-12-myristate-13-acetate-induced protein) for which no associations have previously been reported [5], and 288 kb downstream from MC4R (Melanocortin 4 Receptor). Rs12957347 is correlated with rs12970134 reported in [6] (r^2^ = 0.7) which is 155 kb downstream of MC4R, and gave a p-value = 2.4×10^−5^ in the current study, and therefore is not included in Table 2.

### Detail of association results for each trait

#### HDL-cholesterol

Associations with p<10^−6^ and PPA>0.5 were found at two SNPs near CETP, two SNPs at 8p21.3 near the LPL (Lipoprotein lipase) gene, two SNPs at 11q12.2 near the FADS1 and FADS2 (Fatty acid desaturase) genes, and one SNP at 21q22.3 near FLJ41733. These associations have been reported elsewhere in Indian Asians and Europeans [6], [7], with the exception of FADS1 and FADS2, which have been reported in Europeans [7], [8], but not previously in Indian Asians. The minor allele fractions (MAF) and effect sizes among the Indian Asians in our study are similar to those previously reported in other populations (see references listed in Table 2). The association at 21q22.3 near FLJ41733 has not previously been reported, nor is this locus known to have any metabolic function. A further SNP showed modest association (p<10^−6^, PPA = 0.35) at C11orf10, which maps to 11q12.2 near FADS1/FADS2. An LD table for the HDL-associated SNPs in the above three loci is in Table S2.

#### Type 2 diabetes

SNP rs7903146 near TCF7L2 (p<10^−6^ and PPA = 0.69) is a well-established T2D SNP in Caucasians [9]–[15]. The MAF and effect size among the Indian Asians in our study are similar to those previously reported for Europeans (Table 2).

#### Diastolic blood pressure

SNP rs7865146 is located<3 kb from the Endoglin (ENG) gene. In our study it showed suggestive evidence of association (PPA = 0.57), each copy of the rare allele reducing DBP by 1.19 mmHg (95% CI: 0.71 to 1.67). An association with DBP is biologically plausible: ENG encodes a type I membrane glycoprotein and is part of the TGF-beta receptor complex. It is crucial for maintaining vascular integrity and has a role in the development of the cardiovascular system [16]. Its expression is regulated during heart development [5]. A large meta analysis did not show association with variants within or near ENG in Europeans [17]. Although that study included 12,000 Indian Asians, only 12 SNPs were genotyped in these individuals, none of them near ENG.

## Discussion

The metabolic syndrome and its components are a major health concern, particularly in Indian Asians. The GWAS approach has met with some success in dyslipidemia, type 2 diabetes and obesity phenotypes [4], [7], [15], [17], with most studies to date being conducted in Europeans. Our study has further confirmed a number of previously reported associations, in some cases for the first time in Indian Asians, and identified some novel suggestive associations requiring further confirmation.

The metabolic syndrome consists of a number of phenotypes that tend to co-occur, raising the question of whether or not they have common genetic mechanisms [18], [19]. A number of definitions for the metabolic syndrome have been developed over the years, including those proposed by IDF, NCEP ATPIII or WHO [1], [20], [21]. We chose the IDF definition, which is the most recent and incorporates ethnicity by providing different criteria for the metabolic syndrome in different ethnic groups [1]. Most published associations for the metabolic syndrome are only with individual component phenotypes, or in some cases with multiple phenotypes but not matching any of the above definitions. Joy et al. [22] reviewed a large number of genetic association and linkage studies for the metabolic syndrome using all definitions, and concluded that these studies have not provided any confirmed associations. Our results for Indian Asians also found no evidence for common genetic mechanisms underlying the metabolic syndrome, despite its high prevalence in this population, and the fact that we enriched for extreme metabolic syndrome phenotypes through selective genotyping of the participants.

Bayesian statistical methods are still rarely used in reporting genetic association studies, and we hope that our report will give further illustration of their potential benefits. Firstly, intuition gives little guide as to how important is an association of, say 8×10^−7^, because the answer depends on power which in turn depends on several factors including the MAF. In contrast, a PPA of 0.6 is immediately interpretable irrespective of power or any multiple testing issues. On the other hand, p-values are usually simpler to compute, compared with a PPA that requires assumptions about the distribution of effect sizes [3], and so we have reported both here. We required that associations satisfy both p<10^−6^ and PPA>0.2 to be worthy of reporting in Table 2 (weaker suggestive associations are reported in Table S1). A second advantage of a Bayesian approach is that it has allowed us to give some weight to non-additive genetic associations, while still giving an appropriate weight to the more prevalent additive associations. Using p-values it is not easy for the researcher to control the weight given to different genetic models in an interpretable way. In fact, the associations reported in Table 2 are all consistent with an additive genetic model, and allowing for general associations had little impact on our results. However we were able to allow for this possibility without incurring a multiple testing penalty.

## Materials and Methods

### Ethics Statement

The London Life Sciences Population (LOLIPOP) study is approved by the Ealing and St Mary's Hospitals Research Ethics Committees and written consent was obtained from all participants.

### Participants

Participants were selected from the LOLIPOP study [6], an ongoing collection of phenotypic data and blood samples on a large number of Indian Asian and European white men and women living in West London.

We had available a pool of 8371 men aged 35 to 75 years, for whom all four grandparents were of Indian Asian descent born on the Indian subcontinent. Recruitment and data collection are described elsewhere [6]. The pool was split into two groups of sizes 4100 and 4271, from which 2706 and 2746 were selected for genotyping in stages 1 and 2, respectively. In each stage the same “top and tail” selection procedure was used, based on four traits. All T2D cases on treatment were selected, and other individuals were selected on the basis of having a low glucose level (<5.6 mmol/l), or in the top 500 or bottom 500 for one of waist to hip ratio (WHR), diastolic blood pressure (DBP), or HDL-cholesterol. Alcohol consumption was transformed to a factor with 3 categories: zero, < = 21 units/week, and >21 units/week. Based on significance from multiple regression analysis, we made the following adjustments prior to selection: for non-T2D cases, glucose was adjusted for age and body mass index (BMI); DBP was adjusted for age, BMI and alcohol; WHR was adjusted for age; HDL was adjusted for BMI and alcohol. After adjustment, the individuals with the top and bottom five measurements for each of WHR, DBP and HDL were excluded, to avoid analysing extreme outliers reflecting data anomalies. The top and bottom four glucose measurements in non-T2D cases were also excluded. A summary of the selection procedure is in Table S3. We evaluated a quantitative metabolic syndrome phenotype with a continuous score from 0–5 based on the International Diabetes Federation (IDF) definition summarised in Text S1 [1]. We also considered a binary phenotype with scores 3–5 as cases and scores 0–2 as controls. This did not reveal any new results and is therefore not discussed here.

### Genotyping

The Stage 1 samples were genotyped by DeCode, Iceland, using the Illumina 300HumanHap Bead Chip, which includes 317,968 SNPs of which 308,942 are autosomal. Stage 2 samples were genotyped using a custom array and Illumina Golden Gate technology at Imperial College, Hammersmith Hospital. Due to a problem with genotyping, a random set of 500 samples selected for Stage 2 were not genotyped, and analyses proceeded without these samples. This left a total of 2274 samples genotyped at 1370 SNPs. A summary of the genotyping for both stages is in Tables S4 and S5, and Text S2.

### Statistical analysis

#### I. Quality control

**Figure 2:**
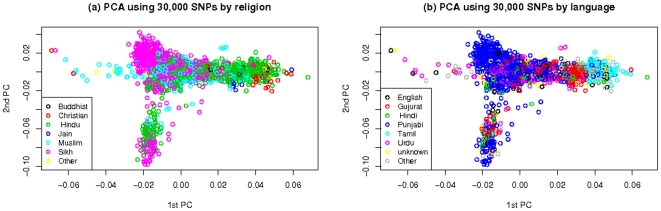
First and second principal components for stage 1 individuals colour coded by (a) religion and (b) language.

Phenotypes and genotype data were subjected to rigorous quality control procedures, detailed in Text S2. In summary, individuals and SNPs were excluded based on genotyping quality, Hardy-Weinberg equilibrium and relatedness. In Stage 1, we investigated the effect of population structure through principal component (PC) analysis. The first two PCs are shown in Figure 2, and indicate a complex pattern of population structure that has some correlation with religious affiliation (Figure 2 top) and language (Figure 2 bottom). Among the first fifteen PCs, only the first four were significantly associated with any phenotype and these four were used to adjust for population structure in the Stage 1 regression analyses. Computation of both PCs and kinship coefficients used only every 10^th^ SNP, in genome order, to minimise any impact of LD. A summary of exclusions is in Table S6.

#### II. Association analysis

In Stage 1, association of individual SNP genotypes with each of the (adjusted) metabolic traits and the metabolic syndrome phenotype were tested in PLINK [23], using logistic regression for T2D and linear regression for the other traits. For each SNP, both additive and recessive genetic models were tested. To maximise power, each individual was included in the analysis of each phenotype, irrespective of the reason for selecting that individual. Perhaps because of this and our adjustments, despite the top-and-tail selection we found that standard regression p-values based on the Gaussian statistical distribution showed correct type 1 error, as evidenced by good adherence of observed quantiles to their null expectations over all but the upper tail of the distribution (Figure S1).

We selected 1536 SNPs for the Stage 2 Golden Gate custom array, based on a number of conditions: 1433 were significant under the additive model, either at p<10^−3^ for one of the four primary phenotypes, or at p<10^−5^ for the two metabolic syndrome phenotype, or at p<10^−5^ under the recessive model for any phenotype. An additional 103 SNPs were selected based on being proxies (r^2^>0.9) for a top-ranked SNP, or for a SNP with low design score, or based on candidate SNPs from the literature. SNPs with p>10^−6^ were excluded if they had high LD (r^2^>0.9) with a genotyped SNP, or low design score. We tested association of Stage 2 SNPs with each of the primary phenotypes and the metabolic syndrome phenotype.

Data from both stages were pooled and association analysis was carried out on the combined data, in the same way as for Stage 1, including testing for recessive and dominant models, except that PCs were not available to adjust for population structure. Bayes factors (BFs) were calculated using both additive and general models. For the quantitative traits, the additive model assumed a linear trend in trait mean and constant trait variance with increasing minor allele count (0,1, or 2), while the general model allowed any changes in both mean and variance over genotypes. For the binary traits (T2D and the two definitions of the metabolic syndrome), the general model is described as BFr in the supplementary material of [3]. Full R code for the quantitative trait BFs is given in Text S3. We used the default prior parameters, which imply that a 95% interval for the mean effect size is approximately +/−0.4 phenotype standard deviations, while under the general model the phenotypic variance was allowed to vary over genotypes by about 5%. The two Bayes factors were combined using Bayes theorem to generate the PPA, giving a 4∶1 weight in favour of the additive model, and a prior probability of association of 10^−4^ at each SNP. See Text S3 for the full R code that includes all parameter values for all four Bayes factors.

## Supporting Information

Text S1 (0.01 MB DOC)

Text S2 (0.02 MB DOC)

Text S3 (0.02 MB DOC)

Table S1 (0.03 MB DOC)

Table S2 (0.01 MB DOC)

Table S3 *Although individuals were selected based on the top and bottom 500 ranked samples, some extra criteria were used in the selection process as set out in the table. These criteria were applied to the raw measurements, whereas selection of the “top” and “tail” was carried out on adjusted traits as described in the methods section.
(0.01 MB DOC)

Table S4 (0.01 MB DOC)

Table S5 *In the combined analyses of stages 1 and 2, the “Total” numbers applied to the quantitative traits (HDL, WHR, DBP and quantitative metabolic syndrome) and the Cases and Controls numbers applied to T2D and binary metabolic syndrome.
(0.02 MB DOC)

Table S6 (0.01 MB DOC)

Figure S1 (0.08 MB TIF)
