# Supplementary material for: A Genome-Wide Association Study of the Metabolic Syndrome in Indian Asian Men
Source: PLoS One. 2010 Aug 4;5(8):e11961. doi: 10.1371/journal.pone.0011961 (PMC2915922; doi:10.1371/journal.pone.0011961)
Supplement: Text S1 — (0.01 MB DOC) [file pone.0011961.s001.doc]

**Text S1**

IDF definition for metabolic syndrome was as follows:

Waist circumference >= 90cm plus any two of the following:

1- Raised triglycerides >= 1.7 mmol/L or specific treatment for this lipid abnormality.

2- Reduced HDL-cholesterol < 1.03 mmol/L or specific treatment for this loipid abnormality

3- Raised blood pressure >= 130mmHg Systolic or >= 85 mmHg Diastolic or treatment of previously diagnosed hypertension

4- Fasting plasma glucose > = 5.6 mmol/L or previously diagnosed type 2 diabetes.
