# Supplementary material for: A Genome-Wide Association Study of the Metabolic Syndrome in Indian Asian Men
Source: PLoS One. 2010 Aug 4;5(8):e11961. doi: 10.1371/journal.pone.0011961 (PMC2915922; doi:10.1371/journal.pone.0011961)
Supplement: Text S2 — (0.02 MB DOC) [file pone.0011961.s002.doc]

**Text S2**

**Genotyping and quality control**

Genotyping methods followed the Infinium 2 Protocol. In conjunction with each SNP genotype, Illumina generates a GenCall score (GCS). The GenCall score is a score designed as a means by which to rank and filter out failed genotypes or DNAs.

Individual genotype missingness indicates DNA quality, and can indicate poor DNA quality. We used a threshold of 5%. SNPs out of HWE (p < 10^-6^) were kept in the analysis and flagged; none of these is reported in Table 2.

Genotypes with GCS <0.6 were relabelled as missing. This is more conservative than Illumina’s recommended threshold of 0.3, and increases the apparent missingness rate. Prior to the combined analysis, data for each stage were filtered sequentially in the following steps: SNPs with average missingness >0.1 were excluded, followed by excluding individuals with average missingness >0.05. Approximately 30,000 SNPs, selected as every 10th SNP in genome order, were used in the estimation of the kinship coefficients and principal components to avoid using blocks of SNPs in high LD. Kinship coefficients between all pairs of individuals, and inbreeding coefficients for each individual, were estimated using standard formulas based on excess allele-sharing averaged over all SNPs. A minimum number of individuals were removed such that no retained pair of individuals had kinship coefficient > 0.175. When a choice was possible, the one with the least missing genotypes was retained. A total of 140 and 172 individuals were excluded in stages 1 and 2 respectively due to sample contamination, presence of duplicates or related individuals (Table S2). In the stage 1 data, significant correlations were found with any of the analysed traits only for the first four principle components, and these were included as covariates in the stage 1 association analyses.

Lambda values, based on the median chi-square that reflects whether the distribution of the p-values conforms to the null hypothesis, were calculated for each of the phenotypes for stage 1 data. Values greater than 1 indicate an elevation of the average chi-square association statistic owing to the presence of population stratification. Lambda values were as follows: HDL-cholesterol (lambda= 1.09), WHR (lambda=1.25), DBP(lambda=1.03) and T2D(lambda=1.03). It was not possible to adjust for population structure in the combined analyses. Although the value of lambda for WHR is high, it has not led to any positive association being reported in Table 2. Further, the value of lambda for HDL-cholesterol was reduced by using a log transform in the combined analyses.

**Power of the study**

We used CaTS [1] and QpowR [2] software for power calculations, assuming a significance threshold of P < 1. 6 x 10^-7^ based on a Bonferroni-corrected family-wise alpha = 5%. Our two-stage design provided 80% power to identify genetic variants with heritability = 0.01 for a quantitative traits, and odds ratio of 1.25 for categorical traits with disease prevalence of 20% assuming an additive model and MAF = 0.3.

**References**

1. Skol AD, Scott LJ, Abecasis GR and Boehnke M Joint analysis is more efficient than replication-based analysis for two-stage genome-wide association studies. Nat Genet (2006) 38:209-13

2. Steibel JP and Abecasis G. QpowR: Interactive power calculator for two­stage genetic association

studies of quantitative traits.

(2008) https://www.msu.edu/~steibelj/JP_files/QpowR.pdf.
