# Supplementary material for: A Genome-Wide Association Study of the Metabolic Syndrome in Indian Asian Men
Source: PLoS One. 2010 Aug 4;5(8):e11961. doi: 10.1371/journal.pone.0011961 (PMC2915922; doi:10.1371/journal.pone.0011961)
Supplement: Text S3 — (0.02 MB DOC) [file pone.0011961.s003.doc]

**Text S3**

**R script for Bayes factors and posterior probability of association**

The following R script was used to compute the Bayes factors under both additive and general models, and posterior probabilities of association for quantitative traits.

####################### Beginning of script####################

# Copyright (C) 2009 David J Balding, Institute of Genetics, University College London

# This program is free software: you can redistribute it and/or modify it under the terms of the GNU General Public License as published by the Free Software Foundation, either version 3 of the License, or (at your option) any later version. This program is distributed in the hope that it will be useful, but WITHOUT ANY WARRANTY; without even the implied warranty of MERCHANTABILITY or FITNESS FOR A PARTICULAR PURPOSE. See the GNU General Public License for more details <http://www.gnu.org/licenses/>.

#------

# version 1.0

# BFdv returns log10(BF), where BF = Bayes factor, for Gaussian quantitative trait data, where H1 allows both mean and variance to change with genotype, with no linear or other assumption about the way in which either mean or variance changes over genotype.

# The phenotype data are standardised (i.e. y is set to mean zero and unit variance) after individuals with either phenotype or genotype missing are removed; genotypes g must be coded as a, a+1, a+2 for some a.

# tau is the prior precision (inverse of variance) of the trait mean, mu (because of standardised data, E[mu] is assumed to be 0). We usually expect the variation of mu over genotypes to be much less than the data variance, so tau>>1. The value tau = 25 corresponds roughly to an assumption that mu has a prior 95% interval from -0.4 and 0.4, whereas tau = 100 implies that this interval is half as wide.

# df and sc are the shape and rate parameters of the gamma prior for the trait data precision (NB mean of gamma = df/sc). Given standardised phenotype data, df = sc is appropriate (so mean = 1). Choosing df = 400 implies a 95% equal-tail interval of (0.90,1.10), so this prior allows the precision and hence the variance to vary over genotypes by about 10%. If tau is doubled, the width of this interval is approximately halved, and vice-versa.

BFdv = function(y,g,df=800,tau=100){

mp = function(x,df,tau){

ndat = length(x); if(ndat == 0) return(0); sc = df

z = sum((x-mean(x))^2) + ndat*tau*mean(x)^2/(ndat+tau)

return(lgamma(df+ndat/2)-lgamma(df) - log(1+ndat/tau)/2 + df*log(sc) - (df+ndat/2)*log(sc+z/2))

# we have removed the constant involving pi because it cancels

}

miss = is.na(y) + is.na(g); y = y[miss==0]; g = g[miss==0] #remove individuals with any missing data

g = g-min(g)+1 # set min g'type to 1

y = (y-mean(y))/sd(y); # standardise y

# y = qnorm(rank(y)/(length(y)+1)) # (optional) replace y values by their Gaussian quantiles, to investigate sensitivity of the BF to the assumption that y is Gaussian.

return(0.4342945*(mp(y[g==1],df,tau)+mp(y[g==2],df,tau)+mp(y[g==3],df,tau)+(length(y)-1)/2)) # the BF is the sum of mp for each genotype minus mp evaluated over all the data (corresponding to the null of no effect of g) and converted from natural log to log10

}

# BFlm returns log10(BF) for Gaussian quantitative trait data, where H1 corresponds to a (homoscedastic) linear regression model. The algorithm uses formula (9.44) in Sec 9.30 p 257 of O'Hagan A, Kendall's Advanced Theory of Statistics, Volume 2B Bayesian Statistics, Edward Arnold, 1994. The terms in (9.44) are defined earlier in Chapter 9. The null model is that the data are iid standard normal.

# We assume standardised phenotype data (i.e. y has mean zero and unit variance). If the three genotypes are coded linearly in allele counts (e.g. as 1,2 and 3) then H1 specifies an additive genetic model, but other codings can lead to other models, e.g. 0,1,1 and 0,0,1 for recessive and dominant models. The genotypes are centred (mean set to 0) so that the regression intercept = 0.

# df is the shape and a the rate parameter of the gamma prior for the precision (inverse of residual variance). We set a = df so that prior mean = 1; this is appropriate for standardised y if there is no effect (slope = 0); a true effect can reduce the residual variance from 1 but the effect will typically be small. The value of df should be similar as for BFdv.

# Vslo is the prior variance of the regression slope parameter under H1, it should typically be similar to 1/tau in BFdv.

BFlm = function(y,g,df=800,Vslo=0.01)

{

miss = is.na(y) + is.na(g); y = y[miss==0]; g = g[miss==0] #remove individuals with any missing data

y = (y-mean(y))/sd(y); g = g - mean(g); n = length(g) # centre the genotypes

y = qnorm(rank(y)/(n+1)) # (optional) replace y values by their Gaussian quantiles, to investigate sensitivity of the BF to the assumption that y is Gaussian.

a = df

# the "s" in variable names corresponds to O'Hagan's "*"

# now evaluate O'Hagan's (9.16) to (9.18) under H1

iVs = 1/Vslo+sum(g*g)

ms = sum(g*y) / iVs

dfs = 2*df + n-1

as = 2*a + n-1 - ms^2 * iVs

return(0.4342945*(-log(iVs)/2-log(Vslo)/2+df*log(2*a)+n/2*log(2)+lgamma(dfs/2)-lgamma(df)-dfs/2*log(as) + (n-1)/2)) # return log10(BF)

}

# compute PPA; wt is the weight assigned to 10^BFdv (general model) relative to that assigned to 10^BFlm (linear regression model); e.g. wt = 0.25 means a 4:1 weighting, which means 80% and 20% relative weights for the two BFs. pri is the prior probability assigned to H0, the null hypothesis of no association; the default value of 10^-4 implies that 300 Kb of the total human genome length of around 3,000 Mb is expected to be associated with the phenotype (this could be in one large LD block or several loci with less LD).

PPA = function(y,g,wt=0.25,pri=10^-4) # computes the posterior probability of association (PPA) under a weighted combination of additive and general models

{

BF = 10^c(BFlm(y,g),BFdv(y,g))

wBF = (BF[1]+wt*BF[2])/(1+wt)

pri*wBF/(1-pri+pri*wBF)

}

####################### END of script ###################################################
