# Supplementary material for: A Genome-Wide Association Study of the Metabolic Syndrome in Indian Asian Men
Source: PLoS One. 2010 Aug 4;5(8):e11961. doi: 10.1371/journal.pone.0011961 (PMC2915922; doi:10.1371/journal.pone.0011961)
Supplement: Table S1 — (0.03 MB DOC) [file pone.0011961.s004.doc]

**Table S1**. SNPs achieving significance at p < 10^-5^ for the combined S1 and S2 data set.

| **Phenotype** | **SNP** | **Gene** | **Chr** | **MAF(S1S2)** | **S1S2 p-value** |
| --- | --- | --- | --- | --- | --- |
| **DBP** | rs7865146 | ENG | 9 | 0.37 | 1.0E-06 |
| **HDL** | rs3764261 | CETP | 16 | 0.36 | 9.8E-48 |
| **HDL** | rs9989419 | CETP | 16 | 0.39 | 4.5E-19 |
| **HDL** | rs2083637 | LPL | 8 | 0.24 | 2.2E-09 |
| **HDL** | rs1535 | FADS2 | 11 | 0.18 | 4.0E-07 |
| **HDL** | rs174546 | FADS1 | 11 | 0.18 | 4.0E-07 |
| **HDL** | rs4523270 | LPL | 8 | 0.27 | 5.1E-07 |
| **HDL** | rs102275 | C11orf10 | 11 | 0.20 | 6.2E-07 |
| **HDL** | rs496300 | FLJ41733 | 21 | 0.16 | 9.0E-07 |
| **HDL** | rs1085093 | GLTP | 12 | 0.45 | 2.7E-06 |
| **HDL** | rs2217332 | HERPUD1 | 16 | 0.19 | 2.9E-06 |
| **HDL** | rs2292354 | GIT2 | 12 | 0.46 | 7.0E-06 |
| **HDL** | rs174556 | FADS1 | 11 | 0.16 | 9.8E-06 |
| **T2D** | rs7903146 | TCF7L2 | 10 | 0.30 | 6.7E-07 |
| **T2D** | rs531676 | CRTAC1 | 10 | 0.45 | 9.3E-06 |
| **WHR** | rs2113334 | ERCC4 | 16 | 0.41 | 2.6E-06 |
| **WHR** | rs9315632 | STOML3 | 13 | 0.28 | 2.7E-06 |
| **WHR** | rs886427 | PKMYT1 | 16 | 0.28 | 6.0E-06 |
| **WHR** | rs2835810 | DYRK1A | 21 | 0.37 | 6.3E-06 |
| **WHR** | rs164898 | ZNF644 | 1 | 0.27 | 6.3E-06 |
| **WHR** | rs817737 | TRPC4 | 13 | 0.20 | 9.5E-06 |
| **Metabolic syndrome** | rs12957347 | PMAIP1 | 18 | 0.36 | 6.8E-06 |
