# Supplementary material for: A Genome-Wide Association Study of the Metabolic Syndrome in Indian Asian Men
Source: PLoS One. 2010 Aug 4;5(8):e11961. doi: 10.1371/journal.pone.0011961 (PMC2915922; doi:10.1371/journal.pone.0011961)
Supplement: Table S2 — (0.01 MB DOC) [file pone.0011961.s005.doc]

**Table S2**. LD information for some of the significant SNPs

|  | **Gene** | **SNP1** | **SNP2** | **D'** | **r2** |
| --- | --- | --- | --- | --- | --- |
| Chr 16 | CETP | rs9989419 | rs3764261 | 0.66 | 0.16 |
| Chr 11 | C11orf10/FADS1/FADS2 | rs102275 | rs174546 | 1.00 | 0.87 |
|  |  | rs102275 | rs1535 | 0.98 | 0.80 |
|  |  | rs174546 | rs1535 | 0.99 | 0.96 |
| Chr 8 | LPL | rs4523270 | rs2083637 | 0.99 | 0.85 |
