# Supplementary material for: A Genome-Wide Association Study of the Metabolic Syndrome in Indian Asian Men
Source: PLoS One. 2010 Aug 4;5(8):e11961. doi: 10.1371/journal.pone.0011961 (PMC2915922; doi:10.1371/journal.pone.0011961)
Supplement: Table S3 — *Although individuals were selected based on the top and bottom 500 ranked samples, some extra criteria were used in the selection process as set out in the table. These criteria were applied to the raw measurements, whereas selection of the “top” and “tail” was carried out on adjusted traits as described in the methods section. (0.01 MB DOC) [file pone.0011961.s006.doc]

**Table S3**. Inclusion and exclusion criteria for individuals selected for genotyping. HT = hypertension; Hx = has history of; Rx = on medication for.

| **Phenotype** | **Inclusion criteria for “top” individuals*** | **Exclusion criteria for “tail” individuals** |
| --- | --- | --- |
| **T2D** | On oral treatment for T2D (DMRx) | - |
| **Glucose** | - | CHD,T2DHx,T2DRx, T2D, glucose >= 5.6 |
| **WHR** | - | CHD,WHR > 0.97, waist > 90cm, BMI > 26 |
| **DBP** | SBP > 160 or DBP > 100 or HTRx | CHD, HTHx, HTRx, HT-related drugs, SBP >= 130, DBP >= 80. |
| **HDL** | HDL < 1 | CHD, cholesterol- drugs, HDL < 1.1 |
