# Supplementary material for: A Genome-Wide Association Study of the Metabolic Syndrome in Indian Asian Men
Source: PLoS One. 2010 Aug 4;5(8):e11961. doi: 10.1371/journal.pone.0011961 (PMC2915922; doi:10.1371/journal.pone.0011961)
Supplement: Table S4 — (0.01 MB DOC) [file pone.0011961.s007.doc]

**Table S4.**  Summary of genotyping for stages one and two.

|  | **Stage 1** | **Stage 2** |
| --- | --- | --- |
| **No. individuals selected (No. in pool)** | 2,706 (4,100) | 2,746 (4,271) |
| **No. individuals genotyped** | 2,693 | 2,274 |
| **Genotyping platform** | Infinium Illumina humanhap300 | Golden Gate custom chip |
| **Number of SNPs on chip** | 317,000 | 1,536 |
| **Number individuals entered analysis after quality control** | 2,554 | 2,240 |
| **Number of SNPs used in analysis after quality control** | 308,011 | 1,476 |
