# Supplementary material for: A Genome-Wide Association Study of the Metabolic Syndrome in Indian Asian Men
Source: PLoS One. 2010 Aug 4;5(8):e11961. doi: 10.1371/journal.pone.0011961 (PMC2915922; doi:10.1371/journal.pone.0011961)
Supplement: Table S5 — *In the combined analyses of stages 1 and 2, the “Total” numbers applied to the quantitative traits (HDL, WHR, DBP and quantitative metabolic syndrome) and the Cases and Controls numbers applied to T2D and binary metabolic syndrome. (0.02 MB DOC) [file pone.0011961.s008.doc]

**Table S5**. Counts of individuals included in the analysis according to the phenotype on which they were selected in stages 1 and 2. For the “Combined” rows, the total numbers included in the analysis are shown, irrespective of how they were selected.

| **Stage** |  | **T2D** | **HDL** | **WHR** | **DBP** | **MetS** |
| --- | --- | --- | --- | --- | --- | --- |
| **Stage 1** | Cases | 471 | 482 | 473 | 478 | - |
|  | Controls | 466 | 464 | 465 | 465 | - |
| **Stage 2** | Cases | 414 | 410 | 409 | 417 | - |
|  | Controls | 421 | 400 | 403 | 391 | - |
| **Combined** | Cases | 885 | - | - | - | 2200 |
|  | Controls | 3427 | - | - | - | 2107 |
|  | Total* | - | 4550 | 4552 | 4549 | 4560 |
