# Supplementary material for: A Genome-Wide Association Study of the Metabolic Syndrome in Indian Asian Men
Source: PLoS One. 2010 Aug 4;5(8):e11961. doi: 10.1371/journal.pone.0011961 (PMC2915922; doi:10.1371/journal.pone.0011961)
Supplement: Table S6 — (0.01 MB DOC) [file pone.0011961.s009.doc]

**Table S6.**  Details of individual exclusions from the analysis.

| **Exclusion of individuals** | **Stage1** | **Stage2** |
| --- | --- | --- |
| Sample contamination | 6 | 0 |
| Duplicates | 1 | 65 |
| Relateds | 133 | 107 |
| **Total** | 140 | 172 |
