# Supplementary figures and images for: A Genome-Wide Association Study of the Metabolic Syndrome in Indian Asian Men
Source: PLoS One. 2010 Aug 4;5(8):e11961. doi: 10.1371/journal.pone.0011961 (PMC2915922; doi:10.1371/journal.pone.0011961)

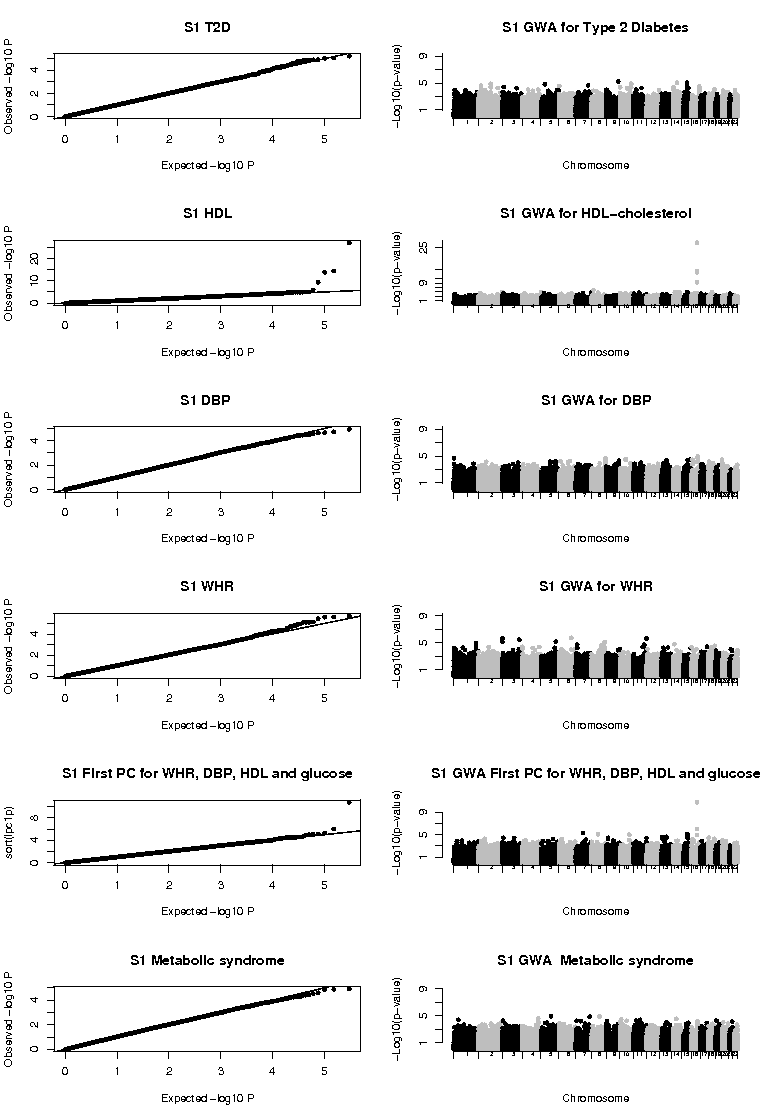

Supplement: Figure S1 — (0.08 MB TIF) [file pone.0011961.s010.tif]
